# Inhibin Inactivation in Female Mice Leads to Elevated FSH Levels, Ovarian Overstimulation, and Pregnancy Loss

**DOI:** 10.1210/endocr/bqac025

**Published:** 2022-03-07

**Authors:** Kelly L Walton, Monica P Goney, Zoe Peppas, Jessica M Stringer, Amy Winship, Karla Hutt, Georgia Goodchild, Shreya Maskey, Karen L Chan, Emilie Brûlé, Daniel J Bernard, William A Stocker, Craig A Harrison

**Affiliations:** 1 Department of Physiology, Monash Biomedicine Discovery Institute, Monash University, Clayton, Australia; 2 School of Biomedical Sciences, The University of Queensland, Brisbane, Australia; 3 Department of Anatomy and Developmental Biology Monash Biomedicine Discovery Institute, Monash University, Clayton, Australia; 4 Department of Anatomy and Cell Biology, McGill University, Montreal, Canada; 5 Department of Pharmacology and Therapeutics, McGill University, Montreal, Canada; 6 Department of Chemistry and Biotechnology, Swinburne University of Technology, Hawthorn, Australia

**Keywords:** inhibin, activin, follicle-stimulating hormone, ovary, folliculogenesis, fertility, ovulation

## Abstract

Inhibins are members of the transforming growth factor-β family, composed of a common α-subunit disulfide-linked to 1 of 2 β-subunits (βA in inhibin A or βB in inhibin B). Gonadal-derived inhibin A and B act in an endocrine manner to suppress the synthesis of follicle-stimulating hormone (FSH) by pituitary gonadotrope cells. Roles for inhibins beyond the pituitary, however, have proven difficult to delineate because deletion of the inhibin α-subunit gene (*Inha*) results in unconstrained expression of activin A and activin B (homodimers of inhibin β-subunits), which contribute to gonadal tumorigenesis and lethal cachectic wasting. Here, we generated mice with a single point mutation (Arg^233^Ala) in *Inha* that prevents proteolytic processing and the formation of bioactive inhibin. In vitro, this mutation blocked inhibin maturation and bioactivity, without perturbing activin production. Serum FSH levels were elevated 2- to 3-fold in *Inha*^*R233A/R233A*^ mice due to the loss of negative feedback from inhibins, but no pathological increase in circulating activins was observed. While inactivation of inhibin A and B had no discernible effect on male reproduction, female *Inha*^*R233A/R233A*^ mice had increased FSH-dependent follicle development and enhanced natural ovulation rates. Nevertheless, inhibin inactivation resulted in significant embryo-fetal resorptions and severe subfertility and was associated with disrupted maternal ovarian function. Intriguingly, heterozygous *Inha*^*+/R233A*^ females had significantly enhanced fecundity, relative to wild-type littermates. These studies have revealed novel effects of inhibins in the establishment and maintenance of pregnancy and demonstrated that partial inactivation of inhibin A/B is an attractive approach for enhancing female fertility.

Inhibin A and B are endocrine hormones produced principally by the testes and ovaries. In female mammals, inhibin A and B are secreted across reproductive cycles in a discordant pattern, with granulosa cells of smaller ovarian follicles producing inhibin B, whereas the dominant follicle and corpus luteum produce inhibin A (1). Inhibin A and B act as negative feedback regulators of follicle-stimulating hormone (FSH) synthesis by gonadotrope cells of the anterior pituitary gland (2). Inhibin B is likely the more important physiological isoform as it suppresses FSH release during the follicular phase of the menstrual cycle (3), and adult males do not produce inhibin A in most species (4, 5). In addition to inhibins, FSH production is titrated by ovarian oestradiol, produced by large developing ovarian follicles in response to aromatase activity (6). In females, FSH regulates cyclic selection of small growing follicles, stimulating their maturation to the preovulatory stage, and promotes inhibin A/B and oestradiol synthesis within granulosa cells (7, 8). Declining ovarian function with menopause onset results in decreased inhibin A/B production and, as a consequence, a rise in circulating FSH (9-11). Clinically, diminished inhibin B or elevations in FSH are indicative of impending ovarian failure (12).

Inhibins are unique heterodimeric members of the transforming growth factor-β protein family of secreted ligands, comprising a common α-subunit disulfide-linked to 1 of 2 inhibin β-subunits (βA in inhibin A, or βB in inhibin B). Initially, the inhibin α- and βA/B-subunits are synthesized in gonadal somatic cells as larger precursor proteins comprising N-terminal pro- and C-terminal mature domains. Following prodomain-guided dimerization (13), the mature inhibin α/β dimers are enzymatically released by proprotein convertases that target consensus RXXR sites (14). Proteolytic maturation is an essential step in inhibin A/B activation, as prodomain retention in non- and partially cleaved inhibin A/B forms results in reduced or no bioactivity (15).

Mechanistically, mature inhibin A and B block the FSH stimulatory activity of activins, homodimers of the inhibin β-subunits. According to current dogma, in the pituitary, activin B binds to type I (ALK4) and type II (ActRIIA/IIB) receptors on the surface of gonadotrope cells, which leads to phosphorylation and activation of SMAD3. Activated pSMAD3 then associates with SMAD4, translocates to the nucleus and, together with FOXL2, binds to the proximal promoter of the FSHβ subunit gene (*Fshb*) (16-19). Transcription of *Fshb* is the rate-limiting step in dimeric FSH synthesis. Inhibin A antagonism of activin is dependent upon interactions with betaglycan, a cell-surface proteoglycan, also known as TGFBR3 (20). Betaglycan binds inhibin A [and inhibin B (21, 22)] directly and promotes the formation of a stable high affinity complex with activin type II receptors (20) to competitively antagonize activin-mediated receptor activation and *Fshb* transcription. Interestingly, a recent study indicates that inhibin B acts preferentially through an alternate gonadotroph-specific co-receptor, termed transforming growth factor beta receptor 3 like (TGFBR3L) to suppress *Fshb* expression (23, 24).

Early studies to understand inhibin physiology utilized mice with a targeted deletion of the inhibin α-subunit gene (*Inha*) (25). *Inha*-deficient mice developed ovarian and testicular sex cord-stromal tumors with 100% penetrance, as early as 4 weeks of age (25). The development of gonadal tumors was rapidly followed by a cachexia-like wasting syndrome, in which mice displayed severe muscle and fat loss, together with anaemia, hepatocellular necrosis, and parietal cell depletion/mucosal atrophy in the glandular stomach (26). While these studies suggested that inhibin A/B had tumor suppressor activities, subsequent experiments indicated that the pathology in *Inha*-deficient mice was caused by pathological increases in activins (27) and gonadotropins (28, 29). As activins, together with gonadotropins, are potent stimulators of granulosa and Sertoli cell proliferation, *Inha*^*−/−*^ mice developed gonadal tumors. The growing tumors produced steadily increasing amounts of activins, which induced significant catabolic effects on many tissues (26). Thus, elevated activin activity in *Inha*^*−/−*^ mice masked the physiological consequences of the loss of inhibins.

Additional attempts to understand inhibin physiology have involved overexpression of the inhibin A subunits in transgenic mice (30-32), administration of recombinant inhibin A (33), or the use of inhibin antiserum. Inhibin A overexpression in female mice reduced circulating FSH and blocked ovarian folliculogenesis at the early antral stage (32). In male mice, elevated expression of inhibin A resulted in reduced testis weights and seminiferous tubule diameter (32). Interestingly, these approaches also identified nonreproductive roles for inhibin A in bone regeneration (31, 33) and suggested that inhibin withdrawal may contribute to accelerated bone loss in postmenopausal women (34). Several groups have also shown that neutralization of inhibin bioactivity using inhibin antiserum can enhance ovulation rates in mice (35) and ruminant animals (36). Although these studies identified target tissues responsive to elevated/reduced inhibin, they did not necessarily reflect inhibin functions at physiological levels. An additional limitation is that for most of these studies only inhibin A, and not inhibin B, was examined.

In this study, we aimed to generate a new mouse model to study the reproductive consequences of inhibin loss of function in the absence of activin overproduction. To this end, we generated mice with an inactivating point mutation (Arg233Ala) in the inhibin α-subunit, which effectively blocked inhibin activity but preserved physiological activin A/B production. Reproductive analyses of *Inha*^*R233A/R233A*^ female mice revealed sex-specific roles for the inhibins in gonadal and reproductive function. Our new model of inhibin loss of function is a valuable research tool for uncovering inhibin A and B physiological activities.

## Materials and Methods

### Generation of Inhibin Mutant Variants

Mammalian expression vectors (pCDNA3.1; Life Technologies, Carlsbad, CA, USA) encoding the human inhibin α- (NM_002191.4), β _A_- (NM_002192.4), and β _B_-subunits (NM_002193.4) were previously generated by our laboratory (37). The inhibin α-subunit vector comprised a polyhistidine tag at the C-terminus of the prodomain to facilitate protein purification. Site-directed mutagenesis was used to silence the cleavage site intervening the inhibin α-subunit pro- and mature domains. An inactivating point mutation (c.694cg>gc/p.232Arg>Ala) was introduced into the inhibin α-subunit using mutagenic primers (sense 5’-catagagcccgagcctcaactcccctg-3’ and antisense 5’- caggggagttgaggccgggctctatg-3’) and the QuikChange Lightning site-directed mutagenesis kit (Stratagene, La Jolla, CA, USA). The resulting plasmid DNA constructs were transformed into XL-Gold Competent *E. coli* (Integrated Sciences, Chatswood, Australia), and colonies were selected and grown in LB media (with 100 μg/mL of ampicillin) at 37°C overnight. DNA was extracted from the cultures using the Wizard Plus SV Miniprep kit (Promega, Madison, WI, USA). Constructs were confirmed by DNA sequencing (Micromon DNA Sequencing Facility, Monash University, Australia).

### Expression Analysis of Inhibin A/B Variants in HEK293 Cells

Inhibin A/B (and activin A/B) protein variants were generated by transient transfection in a human embryonic kidney (HEK293) cell line (Thermo Fisher Scientific, Waltham, MA, USA). In brief, HEK293 cells were plated at a density of 8 × 10^5^ per well in a 6-well plate and maintained in Dulbecco's Modified Eagle media (Thermo Fisher Scientific) with 10% fetal bovine serum (Thermo Fisher Scientific) at 37°C with 5% CO_2_. After 24 hours, each well was transfected with 5 μg (3 μg α-subunit:2 μg β-subunit) of DNA using the transfection reagent Lipofectamine 2000 (Thermo Fisher Scientific) and incubated in 2 mL of serum-free Opti-MEM (Thermo Fisher Scientific) for a further 48 hours, as per manufacturer’s guidelines.

Following transfection, conditioned media samples were analyzed by Western blotting as previously described (38). Inhibin A/B and activin A/B proteins were detected using either an anti-α-subunit monoclonal antibody (R1; Oxford Bio-innovation, Oxford, UK, RRID:AB_2857371; https://antibodyregistry.org/search.php?q=AB_2857371), which binds residues 233–264, an anti-β _A_ subunit monoclonal antibody (E4; Oxford Bio-innovation, RRID:AB_2801574; https://antibodyregistry.org/search.php?q=AB_2801574), which binds residues 401-413 or an anti-β _B_ subunit monoclonal antibody (C5; Oxford Bio-innovation, RRID:AB_2857372; https://antibodyregistry.org/search.php?q=AB_2857372), which binds residues 373-405.

### Inhibin A/B In Vitro Bioactivity Analysis

Sufficient quantities of wild-type and R232A mutant inhibin A/B proteins for in vitro bioactivity assays were generated in HEK293 cells, purified using immobilized metal ion affinity chromatography, and quantified as previously described (38). Inhibin in vitro bioactivity was then determined using an activin-responsive luciferase reporter assay in COV434 cells (38). In short, COV434 cells were first seeded at a density of 75 000 cells per well in 48-well plates and then transfected with an activin-responsive A3-luciferase reporter construct (50 ng/well), the transcription factor FAST2 (100 ng/well), and betaglycan (50 ng/well) using Lipofectamine 3000 (Thermo Fisher Scientific). Following transfection, cells were treated with 150 pM activin A (cat no. 338-AC, R&D Systems, Minneapolis, MN, USA) in the presence of increasing doses of purified inhibins (0-10 nM). The following day, cells were lysed, and luciferase activity was quantified using a CLARIOstar microplate reader (BMG Labtech, Ortenberg, Germany).

### Generation of *Inha*^*R233A/R233A*^ Mutant Mice

*Inha* mutant mice harboring the equivalent to the human R232A point mutation (c.697_699delinsGCG/p.Arg233Ala) were generated by Monash Genome Modification Platform using CRISPR/Cas9-technology on a C57BL/6J background. A guide RNA 5’ GCACGGAGGGAGTTGAACGC 3’ designed to target exon 2 (ENSMUSE00000264874) of murine transcript ID ENSMUST00000037330.5 was coinjected with the repair template (5’GACCACTCCTTTCCTGGTAGCCCACACTAGGGCTCGAGCACCCAGTGCGGGGGAGAGGGCTCGGGCGTCAACTCCCTCCGTGCCTTGGCC TTGGTCTCCTGCGGCTTTGCGCTTGCTGCAGAGGCCT 3’) into mouse zygotes. Following homology directed repair with the injected template, c.697_699delinsGCG nucleotide mutations encoding the p.Arg233Ala codon change was introduced into the genome. A silent mutation c.693C>T was introduced to prevent cutting of the repair template during genome repair. Sequence analysis of polymerase chain reaction products (770 bp) amplified from tail clip DNA, using forward (5’-GGCCATCCCAACACATACGCAG-3’) and reverse (5’-GAGGAGGCGGAGGTGCTTTTAG-3’) primers, followed by a restriction digest with *Hinc*II (resulting in 418 bp and 352 bp products), identified mutant founders to establish the colony. Routine genotyping using tail clip DNA was performed by Transnetyx (Cordova, TN, USA). For analysis, mutant mice were bred to homozygosity and compared with age-matched wild-type littermates.

### Animal Studies

All animal breeding and experimental protocols were approved by Monash University Animal Ethics Committee and conducted in accordance with the relevant code of practice for the care and use of animals for scientific purposes (National Health & Medical Council of Australia). Body composition was monitored weekly using quantitative magnetic resonance (EchoMRI, Houston, TX, USA). At the experimental endpoint, terminal blood samples were collected from anesthetized mice by cardiac puncture for serum hormone assays, and reproductive organs were collected for histological analyses.

### Serum Hormone Assays

FSH levels were measured by an immunofluorometric assay using methods and reagents previously described (39). Serum inhibin A and inhibin B levels were measured using previously established in-house assays (40). Serum levels of activin B and anti-Müllerian hormone were measured using Ansh Lab’s enzyme-linked immunosorbent assay kits (Ansh Laboratories Biotechnology, Webster, TX, USA). Progesterone and testosterone serum levels were quantified using commercially available kits (Immuno-Biological America, Minneapolis, MN, USA).

### Ovarian Histological Analyses

Whole ovaries from 12- or 24-week-old of *Inha*^*R233A/R233A*^ homozygous, *Inha*^*+/R233A*^ heterozygous and wild-type mice were weighed and fixed in neutral buffered formalin for 24 to 48 hours at room temperature. The samples were then processed and paraffin embedded by the Monash Histology Platform. Ovaries were sectioned at 4 µm and every 10th section was stained using periodic acid-Schiff. The stained slides were then imaged using a Brightfield microscope (Zenesis) and follicles counted by a blinded observer. Follicles types examined included primordial (oocyte surrounded by a partial or complete layer of squamous granulosa cells), primary (oocyte with single layer of cuboidal granulosa cells), secondary (oocyte with 1 layer of granulosa cells with no visible antrum), small preantral (oocyte with 1 or 2 small areas of antral fluid), antral (single large antral cavity), corpus luteum (consisting of luteinized follicular mass), or atretic (fragmented oocytes, ruptured/disordered membranes, and scattered granulosa cells), as determined by Myers et al’s guidelines (41).

### Natural and Superovulation Rates

Natural ovulation rates were determined in young (6-8 weeks) *Inha*^*R233A/R233A*^ homozygous, *Inha*^*+/R233A*^ heterozygous and wild-type mice. Female mice were housed overnight with wild-type breeder male studs and inspected the next morning for the presence of vaginal plugs, indicative of copulation. Once mating was confirmed, animals were euthanized, and ovaries and oviducts were collected. Cumulus-oocyte complexes were isolated from the oviducts and mature oocytes were denuded by digestion in M2 media (Sigma-Aldrich, St. Louis, MO, USA) containing 0.3% hyaluronidase (Sigma-Aldrich), and counted on a dissecting stereomicroscope.

For superovulation experiments, young (6-8 weeks) *Inha*^*R233A/R233A*^ homozygous, *Inha*^*+/R233A*^ heterozygous and wild-type mice were intraperitoneally injected with pregnant mares’ serum gonadotropin (5 IU; Prospec-Bio, Rehovot, Israel), followed 44 to 48 hours later by human chorionic gonadotropin (5 IU; Sigma-Aldrich). After 12 to 16 hours, cumulus-oocyte complexes were collected from oviducts and mature oocytes (MII stage) were isolated as described in the previous discussion. Oocytes were graded as MII if they had an intact zona pellucida and evidence of a polar body. Oocytes were classed as abnormal based on intracytoplasmic features (increased cytoplasmic granularity and presence of cytoplasmic inclusions) and extracytoplasmic features (large perivitelline space, perivitelline space granularity, and fragmented or irregular polar body).

### Breeding Experiments

For analysis of fertility, 8- to 12-week-old wild-type, *Inha*^*R233A/R233A*^, and *Inha*^*+/R233A*^ female mice were time-mated with wild-type male stud breeders, and live/dead offspring numbers were recorded at both birth and weaning.

### Pregnancy/Implantation Experiments

*Inha* ^*R233A/R233A*^ homozygous, *Inha*^*+/R233A*^ heterozygous and wild-type females aged 8 to 12 weeks were time mated with aged-matched wild-type male studs and then checked for vaginal plug the following day. Pregnancy was confirmed by visual abdominal examination at 12.5 days postcoitus (dpc). At 17.5 dpc, pregnant females were culled, terminal blood collected, and the uteri and ovaries extracted. The implantation sites were then dissected, and placental and fetal weights were recorded. Maternal ovaries were also fixed with neutral buffered formalin for histological analyses (as previously described).

### Analysis of Male Reproductive Tissues

Testes were dissected, pierced with a fine needle, and immediately fixed in Bouin’s (Sigma-Aldrich) for 5 hours at room temperature. Testes were then processed, cut in half, and embedded into paraffin blocks using standard methods. After embedding, testes were cut at 5-µm sections, dewaxed, and stained using periodic acid Schiff’s reagent and hematoxylin using standard methods. Daily sperm production was measured from whole testes as described previously (42).

### Statistical Analyses

Data were analyzed using unpaired *t*-tests or 2-way analysis of variance with Sidak’s post hoc tests using GraphPad Prism v.9 (GraphPad). Significance was denoted when α (*P-*value) < 0.05. Numerical data are presented as individual values as well as the mean ± standard error of the mean.

## Results

### An Arg^232^Ala Mutation in the Human *INHA* Complimentary DNA Inactivates Inhibin A and B In Vitro

**Figure 1.**
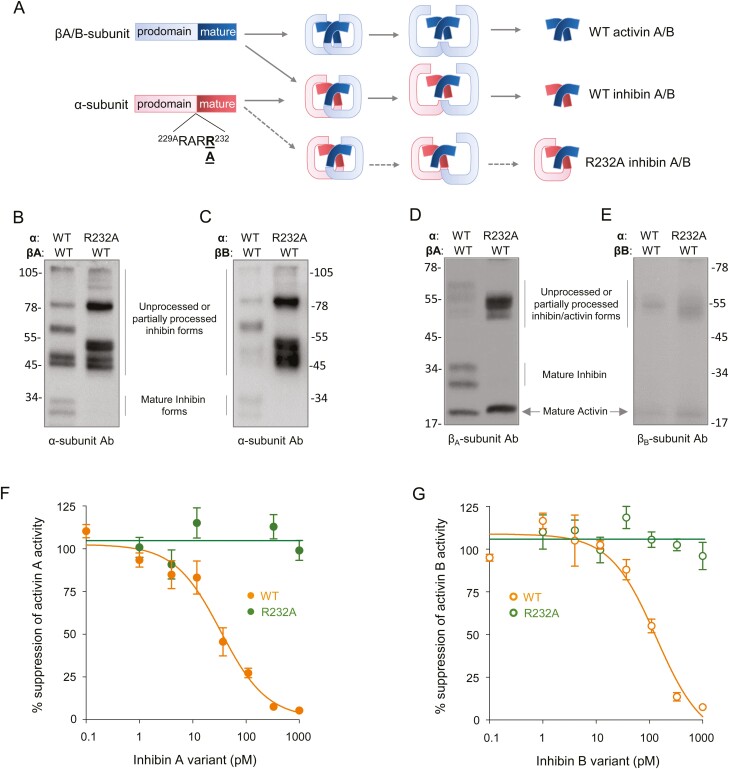
Analysis of the effect of an INHA Arg^232^Ala point mutation on inhibin A/B biosynthesis and in vitro activity. (A) Inhibin A/B are synthesized from α- and βA/B-subunit precursors, which fold and dimerize, before undergoing proteolytic processing to yield the mature bioactive inhibin/activin dimers. The inhibin Arg^232^Ala α-subunit mutation is predicted to block α-subunit (but not β-subunit) processing, resulting in the production of partially cleaved and bio-inactive inhibin A/B. Inhibin α-subunit DNA constructs were co-transfected with either βA- (B, D) or βB-subunit constructs (C and E) into HEK293 cells. Resultant inhibin and activin molecular weight forms secreted into culture media were assessed by Western blot, using antibodies specific to the α- (B-C, R1 Ab), βA- (D, E4 Ab), and βB-subunits (E, C5 Ab). Inhibin A (F) and inhibin B (G) in vitro bioactivities were assessed using a luciferase reporter assay in COV434 granulosa cells. COV434 cells transiently transfected with an activin-responsive luciferase reporter and betaglycan expression vector were treated with increasing doses of inhibin A or inhibin B, and luciferase activity measured (representative result from 3 assay replicates).

The extended inhibin α- and βA/B-subunit precursors undergo processing by proprotein convertases following dimerization, resulting in the generation of mature bioactive inhibin (and activin) A/B dimers (Fig. 1A). Proteolytic maturation is required for ligand activation, as uncleaved inhibin A/B forms have no bioactivity (14). Therefore, we hypothesized that silencing the cleavage site between the pro- and mature domains of the α-subunit, without altering the βA/B-subunit, would specifically inactivate inhibin A/B (Fig. 1A). Here, we introduced a single alanine point mutation at Arg^232^ (R232A) into the human inhibin α-subunit and examined the resulting effects on inhibin and activin synthesis and activity. Western blot analysis on the conditioned media from HEK293 cells co-transfected with inhibin α- and βA/B-subunit complimentary DNA constructs revealed that the R232A mutation did not affect inhibin synthesis overall, as substantial amounts of high molecular weight unprocessed or partially processed inhibin forms were present in the medium (Fig. 1B-1C). The partially processed inhibin A/B forms are the result of the β-subunit being processed normally but the α-subunit remaining uncleaved, owing to the introduced inactivating R232A point mutation. Importantly, the synthesis and maturation of activin A and B were not affected by the R232A mutation in the α-subunit (Fig. 1D-1E). An in vitro reporter assay revealed that the α-subunit R232A mutation abolished the ability of inhibin A or inhibin B to suppress activin signalling in COV434 granulosa cells expressing betaglycan (Fig. 1F-1G). Together, these findings indicate that an R232A mutation in the α-subunit prevents inhibin maturation and blocks in vitro bioactivity, without interfering with activin A/B synthesis.

### Inhibin Inactivation in Mice Results in Elevated Serum FSH but Does Not Cause Cachexia

**Figure 2.**
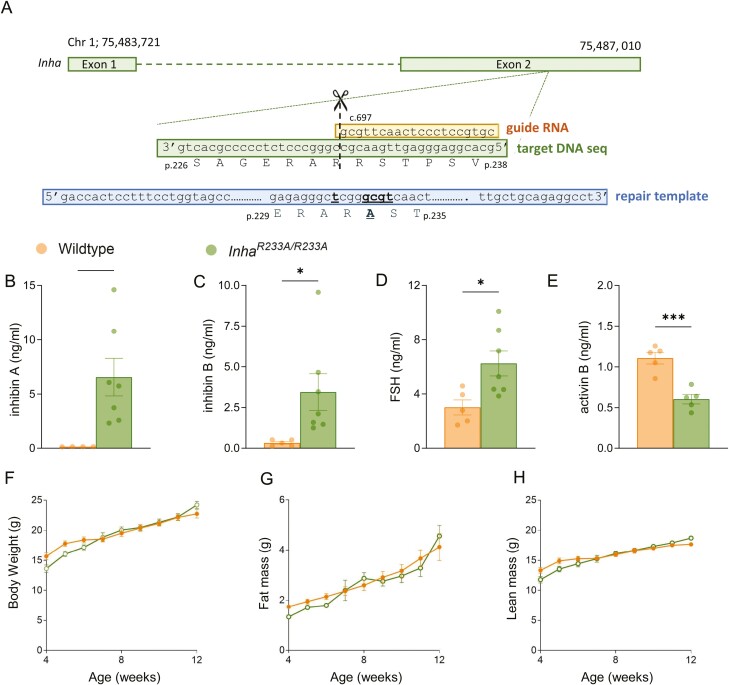
Generation and initial characterization of a genetically modified *Inha*^*R233A/R233A*^ mouse. (A) CRISPR/Cas9-technology was used to introduce the Arg^233^Ala mutation into the *Inha* gene (697_699delinsGCG/p.Arg233Ala) in C57BL/6J mice. A guide RNA designed to target exon 2 of the murine *Inha* gene was coinjected with a repair template into mouse zygotes. Following homology directed repair with the injected template, c.697_699delinsGCG nucleotide mutations encoding the p.Arg233Ala codon change was introduced into the genome. Serum levels of inhibin A (B), inhibin B (C), follicle-stimulating hormone (D), and activin B (E) were measured in 12-week-old female mice by enzyme-linked immunosorbent assay. Body (F), fat (G), and lean mass (H) were monitored weekly by EchoMRI (12 mice per genotype). Bars represent mean ± SE of the mean. * *P* < 0.05, *** *P* < 0.001.

Using CRISPR/Cas9 technology, we introduced an R233A point mutation (c.697_699delinsGCG/p.R233A), which is equivalent to the R232A mutation in human, into exon 2 of the *Inha* gene in C57BL6/J mice (Fig. 2A). At 12 weeks of age, serum inhibin A and inhibin B levels increased markedly in female *Inha*^*R233A/R233A*^ mice (Fig. 2B-2C). However, based on our in vitro analysis, these were likely to be unprocessed/inactive forms of inhibin, which are readily detected in our enzyme-linked immunosorbent assays (40). In support that granulosa cells were producing inactive forms of inhibin A/B, FSH levels in female *Inha*^*R233A/R233A*^ mice (6.3 ± 0.9 ng/mL) were double those observed in wild-type littermates (3.0 ± 0.6 ng/mL) (Fig. 2D). Importantly, circulating activin levels were constrained in *Inha*^*R233A/R233A*^ females (Fig. 2E), with activin B levels actually being significantly lower than in wild-type littermates (activin A was undetectable in both genotypes). Activin B levels may be reduced in 12-week-old *Inha*^*R233A/R233A*^ females as a consequence of increased inhibin formation, but by 6 months of age activin B levels had normalized [Supplemental Figure 1 (43)]. FSH and inhibin A and B remained persistently elevated for up to 6 months in *Inha*^*R233A/R233A*^ females [Supplemental Figure 1 (43)]. In line with normal to low activin B levels, female *Inha*^*R233A/R233A*^ mice did not develop the ovarian tumors and cachexia-like symptoms (Fig. 2F-H) observed in *Inha* knockout mice at 12 weeks of age (25, 27).

### *Inha* ^*R233A/R233A*^ Mice Have Disrupted Ovarian, but Not Testicular, Function

**Figure 3.**
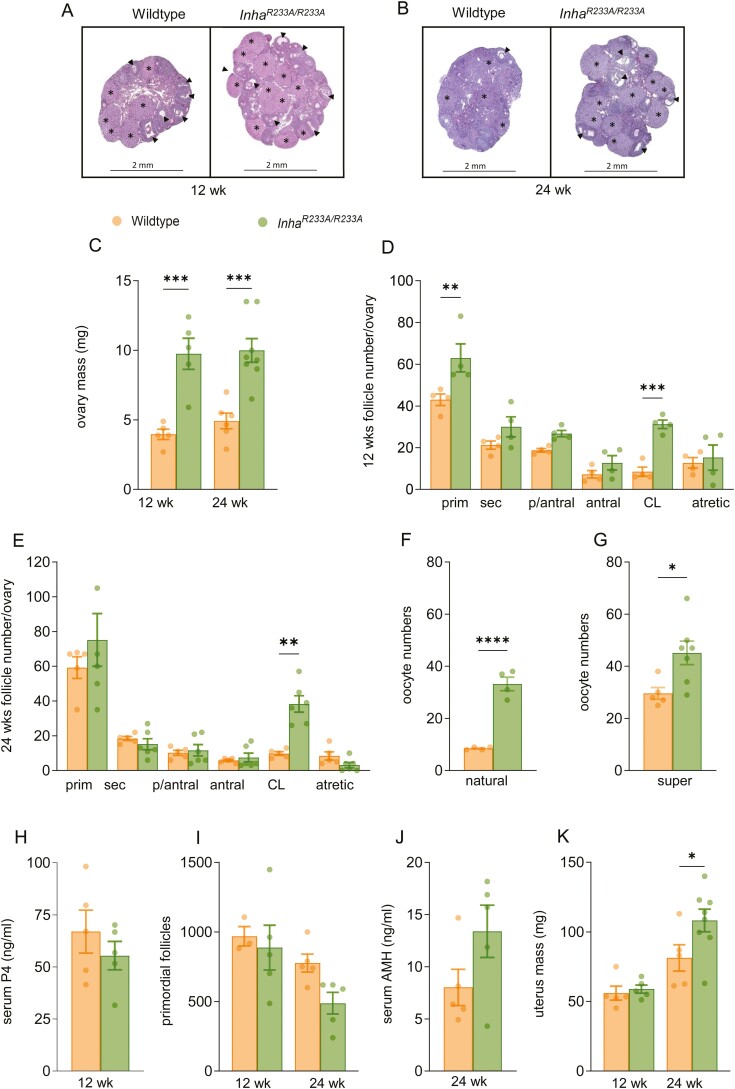
Analysis of ovarian function in female *Inha*^*R233A/R233A*^ mice. Ovaries were harvested from 12- (A) and 24-week-old (B) wild-type and *Inha*^*R233A/R233A*^ mice, weighed (C) and sectioned at 5 µM before being stained with periodic acid-Schiff dye for histological analysis. Primary, secondary, preantral, antral (arrow heads), and atretic follicles, together with corpora lutea (*), were counted in the ovaries of 12- (D) and 24-week-old mice (E). Oocyte numbers following natural (F) and superovulation (G) in 6- to 8-week-old *Inha*^*R233A/R233A*^ mice and wild-type littermates. (H) Serum progesterone levels at metestrus in 12-week-old mice, as measured by enzyme-linked immunosorbent assay (ELISA). (I) Primordial follicle numbers in the ovaries of 12- and 24-week old mice. (J) Serum anti-Müllerian hormone in 24-week-old mice as measured by ELISA. (K) Uteri mass in 12- and 24-week-old mice. Bars represent mean ± SE of the mean. *P < 0.05, **P < 0.01, ***P < 0.001.

Morphologically, ovaries from *Inha*^*R233A/R233A*^ mice appeared normal at both 12 and 24 weeks of age, with follicles at all developmental stages from primordial through to preovulatory, together with corpora lutea (Fig. 3A and 3B). However, ovaries from *Inha*^*R233A/R233A*^ mice were, on average, double the mass of those from aged-matched control female mice (Fig. 3C). Histological analysis supported that the increase in ovarian mass in *Inha*^*R233A/R233A*^ mice was due to these ovaries having significantly more corpora lutea (Fig. 3D and 3E). Indeed, it was found that, on average, there were 4-fold more corpora lutea in the ovaries of both 12- (31 ± 4 vs 8 ± 4 lutea) and 24-week-old (38 ± 11 vs 10 ± 2 lutea) *Inha*^*R233A/R233A*^ mice (Fig. 3D and 3E). The marked increase in corpora lutea numbers in the ovaries of *Inha*^*R233A/R233A*^ mice was attributed to the females ovulating 4-fold more oocytes in natural cycles, compared with controls (33 ± 3 vs 8 ± 1 oocytes/cycle) (Fig. 3F), although the number of atretic antral follicles were not significantly different between genotypes (Fig. 3D and 3E). Further, following hormonal stimulation, *Inha*^*R233A/R233A*^ mice ovulated up to 60 oocytes (average 45 ± 5 oocytes) and significantly more than controls (29 ± 2 oocytes) (Fig. 3G). Interestingly, *Inha*^*R233A/R233A*^ mice ovulated as many oocytes in a natural cycle (33 ± 3 oocytes), as wild-type mice (29 ± 2 oocytes) did following superovulation (Fig. 3F and 3G, Table 1). This supports that the *Inha*^*R233A/R233A*^ mice naturally superovulate, likely owing to more developing follicles proceeding through to ovulation, in response to elevated FSH. Surprisingly, given the increased CL numbers, serum progesterone levels were not elevated at metestrus in *Inha*^*R233A/R233A*^ mice (Fig. 3H). Moreover, heightened ovulation capacity occurred at the expense of oocyte quality in *Inha*^*R233A/R233A*^ mice, as 33% of oocytes (mean 15/45 oocytes per female) retrieved following hormonal stimulation were abnormal, relative to only 14% in controls (mean 4/29 oocytes per female) (Table 1).

**Table 1.** Mean number of oocytes retrieved following superovulation of 6-week-old mice.

Oocytes	Wild-type (n = 5)	*Inha* ^*R233A/R233A*^ (n = 7)
Total	29 ± 2	45 ± 5*
MII	25 ± 1	30 ± 3
Fragmented	4 ± 1	15 ± 3**

Oocytes were retrieved from 6- to 8-week-old mice following hormonal stimulation and graded as either MII or fragmented. Oocyte numbers represent means ± SE of the mean. *P < 0.05, **P < 0.01.

The ovaries of 12-week-old *Inha*^*R233A/R233A*^ mice also contained significantly more primary follicles, possibly indicating accelerated follicle recruitment (Fig. 3C). However, the number of primordial follicles within *Inha*^*R233A/R233A*^ ovaries were not significantly reduced at either 12 or 24 weeks of age, relative to controls (Fig. 3I). Circulating levels of anti-Müllerian hormone, which are indicative of follicle reserve, were also not significantly altered in 24-week-old *Inha*^*R233A/R233A*^ mice (Fig. 3J). Finally, uterus weights were not different between genotypes in 12-week-old females but were significantly heavier by 24 weeks in *Inha*^*R233A/R233A*^ mice, relative to control mice (Fig. 3K).

**Figure 4.**
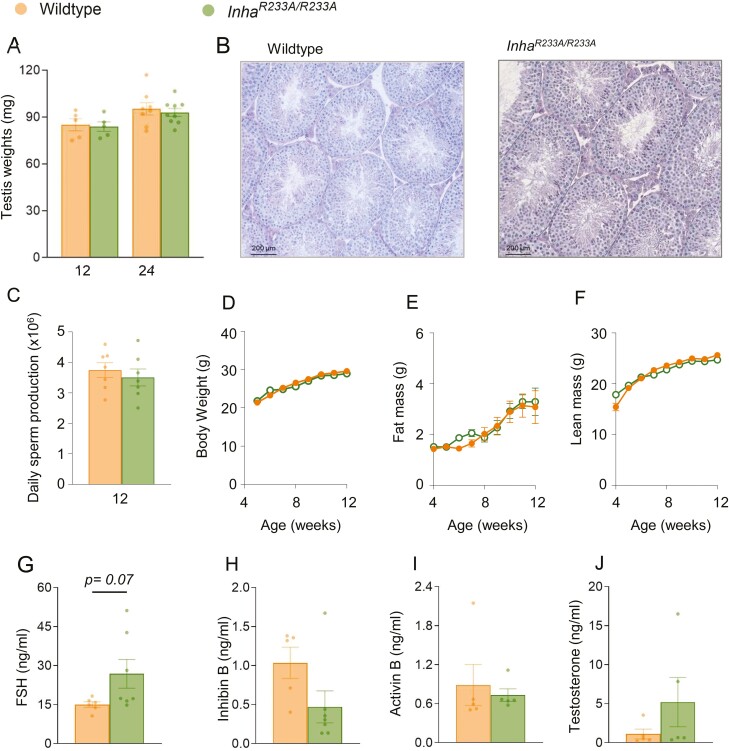
Reproductive characterization of 12-week-old *Inha*^*R233A/R233A*^ males. Testis weights (A), representative histology (B), and daily sperm production counts (C) in wild-type and *Inha*^*R233A/R233A*^ males. Body (D), fat (E), and lean mass (F) were measured weekly by EchoMRI. Serum levels of follicle-stimulating hormone (G), inhibin B (H), activin B (I), and testosterone (J) were measured by enzyme-linked immunosorbent assay. Bars represent mean ± SE of the mean.

Interestingly, the physiological impacts of inhibin loss of function appeared to be sex-specific because testis weights (Fig. 4A), morphology (Fig. 4B), and sperm production (Fig. 4C) were normal in male *Inha*^*R233A/R233A*^ mice. Body composition (Fig. 4D-4F) and hormone levels (Fig. 4G-4J) were also normal in 12-week-old male *Inha*^*R233A/R233A*^ mice, although a near-significant (*P* = 0.07) increase in serum FSH levels was noted and FSH was significantly elevated in 24-weeks-old males [Supplemental Figure 2 (44)].

### Inhibin Inactivation Results in Embryo and Fetal Loss in Female Mice

**Figure 5.**
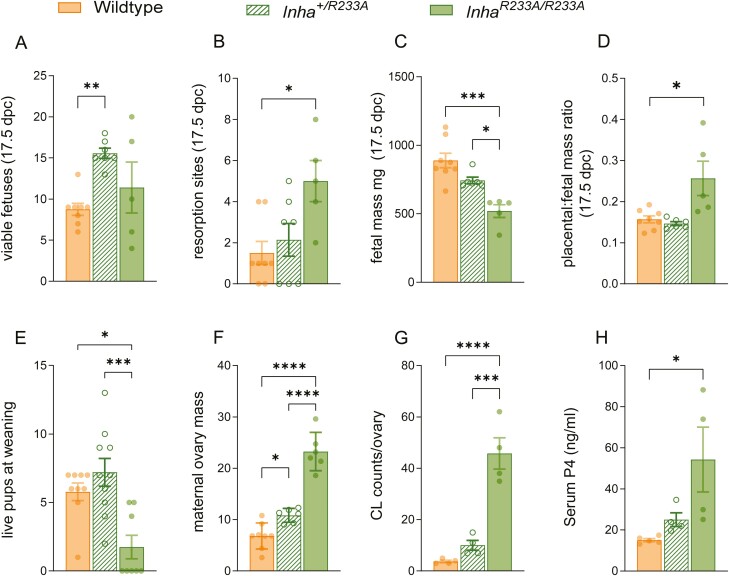
Fertility analysis of female mice. Pregnancy analysis at late gestation (17.5 days postcoitus) in wild-type, homozygote *Inha*^*R233A/R233A*^, and heterozygote *Inha*^*+/R233A*^ female mice showing number of fetuses (A), resorption sites (B), average fetal mass (C), and placental:fetal mass ratio (D) per pregnant dam. (E) Live pups at weaning birthed from female wild-type, *Inha*^*R233A/R233A*^, and *Inha*^*+/R233A*^ dams crossed with wild-type male studs. Maternal ovarian weights (F), corpora lutea counts (G), and serum progesterone levels (H). Bars represent mean ± SE of the mean. *P < 0.05, **P < 0.01, ***P < 0.001, ****P < 0.0001.

Although natural ovulation rates were 4-fold higher in *Inha*^*R233A/R233A*^ mice, compared to age-matched controls (Fig. 3F), there was no difference between the genotypes in the number of fetuses present at 17.5 dpc (Fig. 5A). Moreover, *Inha*^*R233A/R233A*^ females suffered significant embryo/fetal losses, having 3.3-fold more resorption sites at 17.5 dpc compared with wild-type females (Fig. 5B). Of the surviving fetuses in *Inha*^*R233A/R233A*^ females at 17.5 dpc, all were small (516 ± 46 mg), compared to fetuses in control mice (888 ± 53 mg) (Fig. 5C). As a result of these reduced fetal weights, the placental:fetal mass ratio was significantly increased in *Inha*^*R233A/R233A*^ females at 17.5 dpc (Fig. 5D). Not surprisingly, most of these small pups did not survive through to weaning (Fig. 5E). Indeed, of the 67 pups born from matings between *Inha*^*R233A/R233A*^ females and wild-type males, 28.4% were stillborn (19 pups), 34.3% died by postnatal day 5 (23 pups), and 13.4% died by postnatal day 11 (9 pups). Strikingly, only 16 pups (23.8%) survived to adulthood (>12 weeks of age). It is possible that embryo/fetal death secondary to the loss of inhibin function may be attributed to disrupted maternal ovarian function, as the ovaries in *Inha*^*R233A/R233A*^ mice at 17.5 dpc were, on average, 3.5-fold larger than those in control females (Fig. 5F), owing to an abundance of corpora lutea (Fig. 5G). Serum progesterone levels were also significantly elevated in pregnant *Inha*^*R233A/R233A*^ females (Fig. 5H).

### A Reduction in Inhibin Activity Improves Fecundity in Female Mice

Whereas *Inha*^*R233A/R233A*^ females displayed the unusual phenotype of enhanced ovulation rate but reduced fertility, we noted that heterozygote *Inha*^*+/R233A*^ females tended to produce large litters of healthy pups. To understand this distinction, we applied our reproductive analysis to heterozygous mice. Relative to controls at 17.5 dpc, *Inha*^*+/R233A*^ females had almost double the number of fetuses (16 ± 1 vs 9 ± 1) (Fig. 5A) but comparable resorption numbers (Fig. 5B), meaning they carried more pregnancies to late gestation. Fetuses from *Inha*^*+/R233A*^ females (743 ± 24 mg) were smaller than the fetuses from control females (888 ± 53 mg) but significantly heavier than those from *Inha*^*R233A/R233A*^ mice (516 ± 46 mg) (Fig. 5C). The drop in fetal weights in *Inha*^*+/R233A*^ females is likely due to the uterus having to accommodate nearly twice the number of developing fetuses. Placental:fetal mass ratios were also normal in *Inha*^*+/R233A*^ females (Fig. 5D). Finally, there was a (nonsignificant) trend toward more pups birthed from *Inha*^*+/R233A*^ females surviving to weaning, relative to pups birthed from control females (Fig. 5E). Surviving pup numbers from *Inha*^*+/R233A*^ females were significantly greater than from *Inha*^*R233A/R233A*^ females (Fig. 5E). While the ovaries of *Inha*^*+/R233A*^ dams at 17.5 dpc were larger than those in control dams (Fig. 5F), corpora lutea number (Fig. 5G), and serum progesterone levels (Fig. 5H) were comparable to control females.

**Figure 6.**
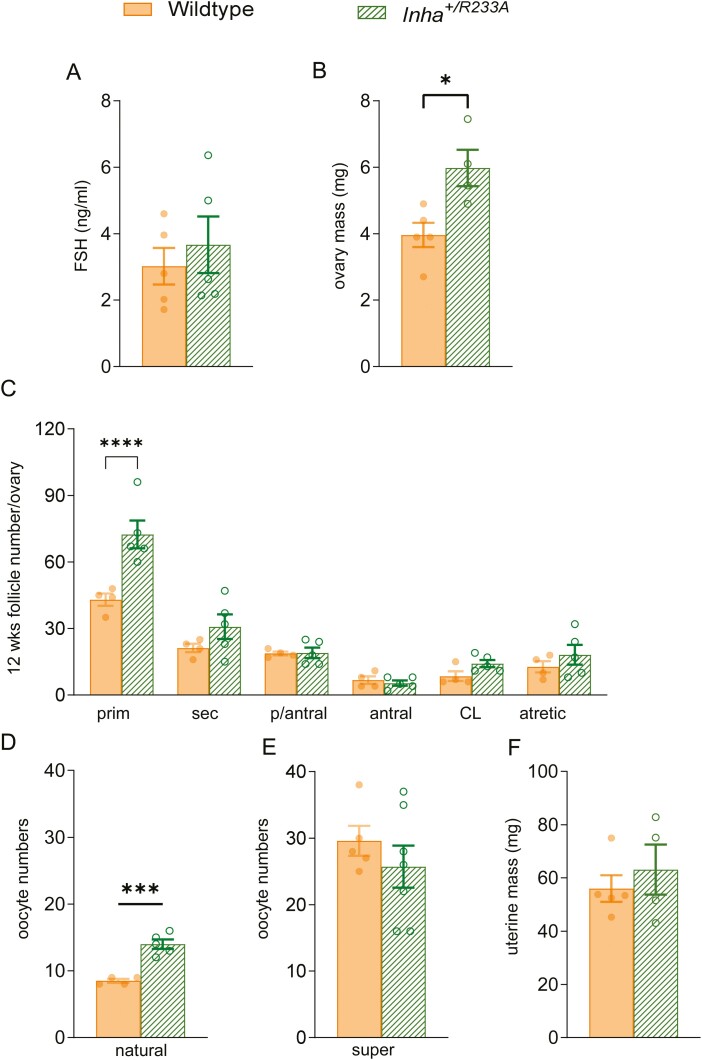
Analysis of ovarian function in heterozygous female *Inha*^*+/R233A*^ mice. In 12-week-old heterozygote *Inha*^*+/R233A*^ and wild-type female mice, serum follicle-stimulating levels were measured (A) and ovaries weighed (B) and sectioned at 5 µM before being stained with periodic acid-Schiff dye for histological analysis. Primary, secondary, preantral, antral (arrow heads), and atretic follicles, together with corpora lutea, were counted in the ovaries of 12-week-old mice (C). Oocyte numbers following natural (D) and superovulation (E) in 6- to 8-week-old *Inha*^*R233A/R233A*^ mice and wild-type littermates. Uteri masses were measured in 12-week-old mice (F). Bars represent mean ± SE of the mean. ***P < 0.001, ****P < 0.0001.

We next sought to understand how partial inactivation of inhibin A/B led to enhanced folliculogenesis and fecundity, by examining the ovarian phenotype in 12-week-old nonpregnant *Inha*^*+/R233A*^ females. It was found that despite heterozygote female mice having normal FSH levels (Fig. 6A), their ovaries were significantly enlarged (Fig. 6B), relative to wild-type littermates. The increase in ovarian mass is likely driven by the near-significant increase in corpora lutea numbers in the heterozygous females (Fig. 6C). Interestingly, it was found that the *Inha*^*+/R233A*^ females ovulated on average 1.6-fold more oocytes in a natural cycle relative to wild-type littermates (14 ± 2 vs 8 ± 1 oocytes/cycle) (Fig. 6D), but the same number of oocytes following hormonal priming (Fig. 6E). While the heterozygote *Inha*^*+/R233A*^ females ovulated less than half the number of oocytes as homozygous *Inha*^*R233A/R233A*^ females (14 ± 2 vs 33 ± 3 oocytes/cycle) in a natural cycle (Fig. 4I), the oocytes from heterozygote *Inha*^*+/R233A*^ females appeared to be developmentally competent. Indeed, on average, >85% of oocytes ovulated from wild-type (mean 8 ovulations and 7 fetuses) or heterozygote mice (mean 14 ovulations and 13 fetuses) resulted in viable fetuses at 17.5 dpc, relative to only 20% of oocytes ovulated from homozygous females (mean 33 ovulations and 6 fetuses) (Figs. 3F, 5A, and 6D). Collectively, our data predict that a partial reduction in inhibin activity enhances fecundity in mice, whereas complete inhibition of inhibin activity reduces embryo/fetal viability.

## Discussion

Over several decades, our groups have significantly advanced molecular understanding of inhibin A/B biosynthesis and activation. We have learned that N-terminal prodomains guide inhibin A/B synthesis (13) and enhance inhibin bioactivity (37). We know that differential glycosylation changes the affinity of inhibin A/B for their classical receptors, betaglycan and ActRII (22), thereby regulating the ability of these isoforms to block activin signaling (45, 46). Recently, we discovered that inhibin B can suppress production of FSH via a distinct co-receptor on pituitary gonadotrope cells (24). In terms of manufacturing, we can now produce inhibin A/B in the absence of contaminating activins, which is a major step toward the development of inhibin therapeutics (38). While these discoveries have greatly advanced our understanding on the mechanisms of inhibin A/B actions, they have provided no new insights into the physiological functions of these hormones.

In this study, we generated a model of inhibin loss of function by introducing a single inactivating point mutation (c.697_699delinsGCG/p.Arg233Ala) into the *Inha* gene. In vitro, this mutation effectively silenced inhibin α-subunit processing, ensuring cells produced no mature inhibin A or inhibin B. Importantly, the Arg^233^Ala inhibin α-subunit mutation did not interfere with homodimerization of the inhibin β _A/B_-subunits to form activins. As such, *Inha*^*R233A/R233A*^ mice did not experience a pathological increase in activin A/B or the subsequent development of gonadal tumors and cachectic wasting, observed in *Inha* knockout mice (27). As a consequence of inhibin inactivation, serum FSH levels were elevated in adult *Inha*^*R233A/R233A*^ female and male mice. Interestingly, this had no discernible effects on testis function in male *Inha*^*R233A/R233A*^ mice. In contrast, in female *Inha*^*R233A/R233A*^ mice, the increase in circulating FSH enhanced follicle development and ovulation rates by as much as 4-fold, resulting in more corpora lutea and, consequently, enlarged ovaries. The increased ovulation rate in *Inha*^*R233A/R233A*^ female mice, however, did not translate to larger litter sizes, owing to increased embryo/fetal resorptions. Moreover, the pups that were born to *Inha*^*R233A/R233A*^ females had low birth weights and most (75%) did not survive to weaning age. Together, this analysis supports that inhibin inactivation results not only in enhanced folliculogenesis and ovulation rates but also pregnancy loss.

Enhanced folliculogenesis and ovulation rates in *Inha*^*R233A/R233A*^ mice are almost certainly owing to the increase in circulating FSH. In support, transgenic expression of human FSH in female mice to levels > 6 IU/L resulted not only in enhanced folliculogenesis and higher ovulation rates but also significant embryo-fetal loss and premature infertility (8). Excess FSH may also trigger an increase in embryo-fetal loss by altering maternal ovarian functions during pregnancy. In support, ovaries in pregnant female *Inha*^*R233A/R233A*^ mice were significantly enlarged, owing to an abundance of corpora lutea, which contributed to elevated serum progesterone during late gestation. Another possibility is that oocyte developmental competence is reduced in the presence of high circulating FSH, as was the case in superovulated *Inha*^*R233A/R233A*^ mice. However, a previous study has shown that the transfer of oocytes from mice expressing transgenic FSH to wild-type recipient dams yielded normal implantation rates and more pups born, compared to controls (47). Thus, a decrease in oocyte quality may require higher gonadotropin levels than the 2- to 3-fold elevation in FSH observed in female *Inha*^*R233A/R233A*^ mice. It is also possible that inhibin inactivation may have auto/paracrine consequences for ovarian function independent of FSH. Indeed, activins have been shown to promote granulosa cell proliferation (48) and regulate ovarian steroidogenesis (49), and intraovarian SMAD2 and SMAD3 activin-targets are required for normal fertility (50).

Interestingly, during the revision of this manuscript, Brûlé et al published a study that strongly supports our findings (23). The authors identified a novel, gonadotrope-restricted inhibin B co-receptor, TGFBR3L. Female mice lacking pituitary expression of both TGFBR3L and betaglycan had high serum FSH, significantly larger ovaries due to marked increases in antral follicles and corpora lutea, and naturally ovulated more oocytes. However, double knockout females did not produce live offspring in breeding trials (23). The phenotypic congruence observed when inhibin A/B are inactivated or when the inhibin receptors are deleted in the pituitary indicates that inhibin control of FSH levels is important during both folliculogenesis and pregnancy.

Intriguingly, while complete loss of inhibin A/B activity led to reduced embryo/fetal survival in homozygous female *Inha*^*R233A/R233A*^ mice, partial loss had the opposite effect. Indeed, compared to wild-type females, pregnant *Inha*^*+/R233A*^ mice carried twice as many fetuses to late gestation (17.5 dpc) and trended toward having more live pups at weaning. The phenotype in the *Inha*^*+/R233A*^ mice is akin to that observed in mice lacking pituitary expression of either betaglycan or TGFBR3L (24). Conditional knockout of betaglycan in pituitary gonadotropes suppressed inhibin A but not inhibin B bioactivity and resulted in more implantation sites (and live pups per litter), owing to enhanced folliculogenesis and ovulation (24). Deletion of the inhibin B co-receptor, TGFBR3L, similarly enhanced ovarian folliculogenesis and fertility (23). Thus, a partial reduction in inhibin A/B activity (via reduced processing or limited receptor availability) appears advantageous for female fecundity. Interestingly, fecundity improvements in female *Inha*^*+/R233A*^, betaglycan knockout, or TGFBR3L knockout mice occur in the absence of discernible increases in circulating FSH (23, 24). However, we suspect that the heightened ovarian function may still be attributed to enhanced FSH tone, either by favoring production of more bioactive FSH glycoforms (51) and/or increased sensitivity of developing follicles to FSH (via upregulation of the FSH receptor).

Based on our findings, it is likely that early inhibin immunization studies in sheep, cows, pigs, and goats were successful in improving female fertility and fecundity because they only partially suppressed inhibin (36, 52-57). Importantly, we now have better tools at our disposal (eg, antibodies and soluble receptors) to reduce inhibin A/B bioactivity and enhance implantation rates and offspring numbers in female mammals. Such technologies could be game changers for global livestock breeding programs, which are currently reliant on restricted supplies of pregnant mare’s serum gonadotropin (or equine chorionic gonadotropin) for superovulation practices. Additionally, such inhibin-targeted technologies may allow for more controlled ovarian stimulation in women undergoing in vitro fertilization.

In conclusion, we generated a new mouse model of inhibin loss of function. Analyses revealed that complete inhibin inactivation results in an elevation in circulating FSH, which triggers ovarian overstimulation and pregnancy loss. Conversely, a partial reduction in inhibin levels enhances female fecundity. Ongoing studies are examining the extra-gonadal impacts of inhibin inactivation, which is of high relevance to postmenopausal women experiencing inhibin withdrawal.

## Data Availability

All data generated or analyzed during this study are included in this published article or in the Figshare data repository listed in the references.
